# Medical Officers of the Navy

**Published:** 1843-04-29

**Authors:** 


THE MEDICAL EXAMINER.

PHILADELPHIA, APRIL 29, 1843.

### MEDICAL OFFICERS OF THE NAVY.

It is not unfrequently asked, what is the mode of obtaining the appointment of assistant surgeon in the navy? but few inquire whether the situation of an assistant surgeon in the navy is really a desirable one. What is the nature of the services required at the hands of this class of officers? how are they cared for on board ships of war? and what is the position of assistant surgeons relatively to other officers in the navy? are questions rarely put by those who quit the University or College, eagerly desiring "to see the world," and commence the career of life. And if they should prudently ask for information on these points, there are few, perhaps, who are competent to give it. It may be interesting to the profession generally to understand this subject; and it may be useful to those who may think of pursuing their professional career in the navy.

The usual manner of obtaining a commission as an assistant surgeon in the navy, is to address an application to the Secretary of the Navy, accompanied by respectable testimonials that the candidate "possesses the *moral* and *physical* qualifications requisite for filling creditably the responsible station, and for performing ably the arduous and active duties which will be required of him." The rule of the Navy Department on this subject requires that applicants must be citizens of the United States—(it ought to be *natives*)—over twenty-one, and not over twenty-eight years of age.

The law provides that "no person shall receive the appointment of assistant surgeon in the navy of the United States, unless he shall have been examined and approved by a board of naval surgeons, who shall be designated for that purpose by the Secretary of the Navy."

Boards of examination are appointed only at such times as the wants of the service render necessary. They usually consi[illegible] of five experienced surgeons of the navy. The Secretary of the Navy selects from the applicants the number of individuals to be examined. To the persons thus selected, "permissions" are given to present themselves to the Board of Examination, stating the time and place of its meeting. By the instruction of the Navy Department, "the Board rigidly scrutinizes the pretensions of each candidate; taking into consideration his physical qualifications and moral habits, as well as his professional acquirements and general knowledge; and reports favourably upon no case admitting of a reasonable doubt, *as the health and lives of the officers, seamen and marines, are objects too important to be committed to ignorant or incompetent hands.* The Board reports the relative merit of the candidates, as shown by the examination; and those of whose qualifications the Board is satisfied, are appointed assistant surgeons as their services are required:" of course, those at the head of the list being first commissioned.

"Candidates of whom the Board makes an unfavourable report are allowed a second examination, at a future session, if they desire it; when, if they again fail, their names are dropped from the list of applicants.

"No allowance is made for the expenses of persons undergoing these examinations, as they are indispensable prerequisites to appointment."

In its decisions the Board is uninfluenced by considering of what school the candidate is a graduate, or whether he has graduated at all: provided he possesses the requisite qualifications, it matters very little where, or by what means, (provided always they are honest,) he has obtained his knowledge. Usually, each member of the Board is especially responsible for one or more branches, but is not confined in his examination to these branches, because he has to give his vote as to whether the candidate is qualified, in all respects, to discharge the duties of an assistant surgeon in the navy, and not as to whether he is, or is not, acquainted with a particular branch of medical science. Each candidate writes a thesis, the subject of which is assigned by the Board. He is given a single sheet of paper, and performs his task in an adjoining apartment. This is a close test of primary education, of readiness, and of the character of the candidate's professional mind; and affords the same indication of ability as an off-hand letter upon a professional subject. That part of the examination which relates to bandaging and the treatment of fractures is generally practical; the candidate being required to exhibit his knowledge of the subject by applying dressings to a mannakin or casts, which are provided for the purpose. In short, the chief object of the examination is to ascertain, if possible, whether the candidate is likely to be an active and efficient practitioner of medicine and surgery.

Having passed this ordeal, the candidate, in the course of time, receives from the President of the United States, "by and with the advice and consent of the Senate," a commission, the tenor of which is precisely like that of every commission officer of the navy.*

For the services of a person thus qualified, the government pays annually $950, when employed on shore; when employed at sea, $1023; and when on leave of absence or waiting orders, $650. These sums include "rations," and perquisites of every kind and description, except an allowance of ten cents per mile when travelling in the United States by order of the government. Like every other officer in the navy, whether on duty or not, the assistant surgeon pays an annual tax of two dollars and forty cents a year to the "Navy Hospital Fund." These sums only are received; and out of them the medical officer is, of course, obliged to subsist himself, provide his uniforms and clothing, and, should he be married, support his family.

Taking the ordinary run of service for five years, sea and shore duty, and a short leave of absence, the average annual pay of an assistant surgeon will be about $930, but not more.

The moment a physician accepts the commission of an assistant surgeon he ceases to be a free citizen, and becomes subject to an aristocratic-military government, in which the position or rank of every individual under its power is defined and established by rule or usage, and he is every hour made to feel that he has many superiors in the new community of his choice.

By those who are unaccustomed to the influence of defined rank, and by those who defer only to individual attributes, the value of rank will not be readily appreciated, because they are not aware that rank, in the naval community, controls association, and points out, precisely, where the foot may, or may not rest, on board of public vessels. Rank is the foundation of power, of personal rights and privileges, and, consequently, of discipline in all military governments.

What is the rank of assistant surgeons in the navy? They have no rank whatever. They are in the anomalous situation of holding a military commission, which secures them no personal rights or privileges; and they are preceded by all warrant officers, except boatswains, gunners, carpenters and sailmakers, and by all teachers of navigation, and steam engineers. Assistant surgeons in the army rank correlatively as masters in the navy, or first lieutenants in the army, and, after five years' service, as captains in the army, or lieutenants in the navy.

In a system of "Rules and Regulations for the Navy," recently submitted to Congress for approval, it is proposed that assistant surgeons shall rank with passed midshipmen, (a grade of sea-officers corresponding with second lieutenants or ensigns in the army,) but stipulates that, on all occasions, "the sea-officers shall take precedence" of them.

And, it may be asked, how are assistant surgeons affected by not having any rank?

Follow the assistant surgeon on board ship, and we shall soon find how very uncomfortable he is made by not possessing a proper and defined rank. He is accommodated in the steerage with the midshipmen, that is, boys of from fourteen years of age and upwards; he sleeps in a cot, (a sort of hammock stretched on a frame,) hung up at night in an apartment of perhaps twenty feet square, with eight or ten hammocks, one or more of which he must press to one side, perhaps, before he can reach his bed. His clothes and books are stowed in a "locker," which, on shore, would be a narrow closet; and, when not professionally employed, if he possess the

* Captains, Commanders, Lieutenants, Surgeons, Assistant Surgeons, Chaplains, and (since the year 1812, prior to which they were warrant officers,) Pursers, are commissioned officers. Marine officers are also commissioned.

Masters, Second Masters, Masters' Mates, Midshipmen, (whether passed or not,) Boatswains, Gunners, Carpenters and Sailmakers, are warrant officers; that is, they are appointed by the Secretary of the Navy without the advice of the Senate. Professors of Mathematics, Teachers at Naval Schools, and Steam Engineers, are also appointed by the Secretary alone. Secretaries and Clerks receive their appointments, which are temporary, from Commanders-in-chief of squadrons, Commanders of vessels, &c.

power of abstraction, he may read and study as much as he pleases, amidst the ordinary Babel-sounds of a crowded steerage. If he serve in a frigate he is somewhat better off, because there he will have a square hole, called a state-room, in the cockpit, which, being below the water line, is totally dark, except when artificially lighted; but when it is remembered that the cockpit is a sort of common vestibule to the store-rooms of the purser, master, marine officer and captain, from which emanate smells of various dietetics kept for the captain's table, it may be understood that the accommodation is even here not of the most eligible kind. On board of ships-of-the-line his condition is not always improved.

The quarter deck in vessels of war, as well as in others, is the sacred part of the ship. A middle longitudinal line divides it into the right, or *starboard side*, and left, or *larboard* side. The starboard side is the side of honour or distinction, and is the privileged promenade of all commission officers, except assistant surgeons, who are permitted to use the larboard side, which is the ground of the midshipmen. In frigates, assistant surgeons are prohibited from using the same ladder or stair to ascend from the lower to the upper deck; there being one ladder provided for the use of the ward-room officers (lieutenants, surgeon, purser, &c.) and captain, and another common to all other officers.

When ward-room officers leave the vessel or return to it, they do so by the starboard side, and their ingress or egress is always accompanied by the ceremony of "two side boys and piping the side." And at night they answer to the sentry's hail, as they approach the ship, "ay! ay!" and are received by two lanterns, to enable them better to see their way into the ship. But assistant surgeons and warrant officers, except the master, leave and return to the ship by the larboard side, and without the ceremony above mentioned; and when they approach the vessel at night, their answer to the sentry's hail is "no! no!" which has been facetiously construed, "nobody, nobody," and they are permitted to get on board in the dark in the best way they can. The starboard and larboard gangways, or points of entrance and exit, may be compared to the front and back entrance of a mansion; the starboard side or front being for the admission of persons of consideration, and the larboard for the admission of individuals of an inferior order, such as servants and others.

Officially, assistant surgeons are held responsible for the execution of the orders given by the surgeon; and they perform the minor operations, as bleeding, leeching, tooth drawing, cupping, and, at least, superintend the mixing and exhibition of medicine in all forms; attend to the cleanliness of the store-room, dispensary and sick-bay; and in the absence of the surgeon, the senior assistant performs all the duties that devolve upon him. If the ship has a competent surgeon's steward, the details are performed by him, under the general superintendence of the assistant.

At the end of five years the assistant is eligible to examination for promotion, and again appears before the Board. The second examination differs somewhat from the first, and the candidate is expected to exhibit a knowledge of the opinions of medical and surgical writers, and show that he has obtained information on nautical hygiene and nautical medicine. If he be found qualified for promotion, his pay is increased. When on leave of absence, his annual pay is $850; when employed on shore, $1150; and at sea, $1273; but neither his condition afloat, nor his duties, are changed in any respect. As a passed assistant surgeon he will probably remain four years before he receives a surgeon's commission.

For the first five years after promotion his annual pay, when on leave of absence, is $1000; when employed on shore, $1250; and when at sea, $1406. But his condition afloat is improved, for he enjoys the personal comforts of ward-room officers, and the same observances of etiquette, to a very considerable extent. Yet he has no rank; and even if he has been a surgeon a quarter of a century, he must give precedence to all lieutenants, although their commissions may be no more than a day old, as well as to the purser, be he ever so young in the service.

If a surgeon of twenty years standing—that is, after he has been twenty-nine years in the navy, including the time he serves as assistant and passed assistant surgeon—go to sea as surgeon of the fleet, the only improvement in his circumstances is, that his annual pay is $2773, for the time he is so employed. If, at the end of the cruise, he is put on leave of absence, he receives $1800 a year; and if employed on shore, $2250; or should he be so fortunate as to receive the appointment of Chief of the Bureau of Medicine and Surgery, he can reside at Washington, and receive $2500 a year while he is working very hard.

Supposing, then, that an assistant surgeon receive a commission when he is twenty-one years of age, he must be fifty before he obtains the very moderate salary of $2250, while in charge of a hospital or other shore station.

Is such a sum, gained under such circumstances, worthy the laudable ambition of a well educated physician or surgeon? Is it a fair remuneration for the services rendered? Will well educated physicians be contented, and serve under such circumstances, unless their relative position in the navy be improved? It must be constantly borne in mind, too, that medical officers share equally with others the discomforts of sea-life, and are liable to the same wear and tear of constitution and privations entailed by absence from country and home consequent upon it, with others.

May we now ask whether the profession does not open a brighter prospect, under other circumstances than the navy affords, for well educated physicians and surgeons? If it does not, why do young men enter it? The government has more than once declared that it is "well aware of the great importance to the navy of a medical corps possessing high professional qualifications," and that it feels "great gratification in believing that, in this respect, no other service surpasses, if it equals, our own." Yet it takes no means to render the condition of medical officers in the navy more tolerable; but permits them to suffer unkindness and degradation from that class of officers which habitually arrogates to itself all power and all privilege of every kind.

Commanding officers, heretofore, have even possessed the power, which they have frequently exerted, of controlling the supply of medicines for the naval service, both as to kind and quantity. And not very long since a medical officer was actually cashiered by a court martial, because he differed with his commanding officer as to the necessity of having a new pestle and mortar in his dispensary—the circumstance being converted into an official charge of disrespect and insubordination.

At the late session of Congress a system of rules and regulations for the government of the navy, drawn up by a captain, a commander, a lieutenant and a purser, (but no surgeon,) under instructions from the Navy Department, was submitted for approval. In his letter accompanying these Rules and Regulations to Congress, the Secretary of the Navy says:

"As officers of acknowledged merit in the different grades of the service were engaged in this duty, (devising rules and regulations,) the fact that the rules and regulations now presented are approved by *all* of them, affords a strong presumption that they are right."

Notwithstanding this, this system of rules &c. provides that commanding officers shall control the supply of medicines, inspect the surgeon's journal, and requires assistant surgeons "to inspect and report, daily, to the executive officer of the ship, the state of the galley," that is, the cooking utensils of the ship's cook!

Such incongruous provisions in a code of laws proposed for approval, would afford to most citizens "a strong presumption that they are" wrong—no matter what might be the reputed intelligence and merit of the persons who devised them.

If space were permitted to contrast the situation of medical officers in the navy with that of pursers and others, as regards pay, or with the army, the case would not be improved.

